# Informing antimicrobial management in the context of COVID-19: understanding the longitudinal dynamics of C-reactive protein and procalcitonin

**DOI:** 10.1186/s12879-021-06621-7

**Published:** 2021-09-08

**Authors:** Damien K. Ming, Ashleigh C. Myall, Bernarnd Hernandez, Andrea Y. Weiße, Robert L. Peach, Mauricio Barahona, Timothy M. Rawson, Alison H. Holmes

**Affiliations:** 1 Centre for Antimicrobial Optimisation, Hammersmith Hospital, Imperial College London, Du Cane Road, London, W12 0NN UK; 2 National Institute for Health Research Health Protection Research Unit in Healthcare Associated Infections and Antimicrobial Resistance, Imperial College London, Hammersmith Campus, Du Cane Road, London, W12 0NN UK; 3 School of Informatics, University of Edinburgh, Scotland, UK; 4 School of Biological Science, University of Edinburgh, Scotland, UK; 5 Department of Neurology, University Hospital of Würzburg, 97080 Würzburg, Germany; 6 Department of Mathematics, Imperial College London, London, UK

**Keywords:** Bacterial co-infection, COVID-19, Biomarkers, Antimicrobial stewardship, Risk stratification, Clinical decision-support

## Abstract

**Background:**

To characterise the longitudinal dynamics of C-reactive protein (CRP) and Procalcitonin (PCT) in a cohort of hospitalised patients with COVID-19 and support antimicrobial decision-making.

**Methods:**

Longitudinal CRP and PCT concentrations and trajectories of 237 hospitalised patients with COVID-19 were modelled. The dataset comprised of 2,021 data points for CRP and 284 points for PCT. Pairwise comparisons were performed between: (i) those with or without significant bacterial growth from cultures, and (ii) those who survived or died in hospital.

**Results:**

CRP concentrations were higher over time in COVID-19 patients with positive microbiology (day 9: 236 vs 123 mg/L, p < 0.0001) and in those who died (day 8: 226 vs 152 mg/L, p < 0.0001) but only after day 7 of COVID-related symptom onset. Failure for CRP to reduce in the first week of hospital admission was associated with significantly higher odds of death. PCT concentrations were higher in patients with COVID-19 and positive microbiology or in those who died, although these differences were not statistically significant.

**Conclusions:**

Both the absolute CRP concentration and the trajectory during the first week of hospital admission are important factors predicting microbiology culture positivity and outcome in patients hospitalised with COVID-19. Further work is needed to describe the role of PCT for co-infection. Understanding relationships of these biomarkers can support development of risk models and inform optimal antimicrobial strategies.

**Supplementary Information:**

The online version contains supplementary material available at 10.1186/s12879-021-06621-7.

## Highlights

- Quantifying risk of bacterial co-infection in COVID-19 is clinically challenging
- 58/207 (28%) of admitted patients had positive microbiology during admission
- Higher CRP levels over time are associated with positive microbiology
- Levels of CRP and trajectory are associated with in-hospital mortality
- Dynamics of biomarkers over time can support infection management

## Background

COVID-19 causes severe illness in a proportion of infected individuals, resulting in an acute inflammatory syndrome with a wide spectrum of presentation. Identifying individuals with concurrent bacterial infections is a major challenge in management. There is a small but growing body of evidence regarding co-infection [1] and clinical evaluation is difficult given high inflammatory burden, extensive consolidation on radiology and a lack of tools with adequate specificity [2]. These diagnostic uncertainties, coupled with prolonged hospitalisation contribute to increased use of empirical antimicrobial therapy [3]. There is a real risk that the ongoing global COVID-19 pandemic may drive increased antimicrobial resistance [4] in acute care and a need for tools to guide optimal antimicrobial prescribing and stewardship.

The likelihood/diagnosis of infection, guided by microbiological sampling and culture, affects decision-making in antimicrobial use as does assessing severity of disease and risk of death. Broader-spectrum antimicrobial coverage is often implemented in severe disease, as captured in WHO guidance for COVID-19 management [5]. Identifying patients at low-risk of bacterial infection and low-risk of clinical deterioration and death would therefore support antimicrobial de-escalation strategies.

In this study we examine the longitudinal dynamics of blood biomarkers for hospitalised patients with COVID-19. C-reactive protein (CRP) and procalcitonin (PCT) are commonly used to differentiate between bacterial infections with other inflammatory conditions [6], but it is unclear how this can be applied to COVID-19 to guide antimicrobial management. It has been shown that initial admission [7] and cut-off values of PCT and CRP predict mortality [8, 9]—however biomarker trends over time remains poorly understood. We therefore hypothesise that their evaluation post onset of COVID-19 symptoms and hospitalisation can offer insights to guide clinical management.

## Methods

The study NHS Trust represents a collection of tertiary hospitals in North-West London, UK with a catchment area of one and half million people. Inclusion criteria into the study were adult patients (> 18 years old) hospitalised in general wards and critical care settings between 1st March and 6th May 2020 who had tested positive for SARS-COV-2 polymerase chain reaction (AusDiagnostics, United Kingdom) through nasopharyngeal swabs, collected by healthcare staff. Indications for SARS-COV-2 testing during this period were hospitalisation with symptomatic infection clinically consistent with COVID-19 infection. The study period reflected a time where SARS-COV-2 testing did not include the testing of healthcare staff and asymptomatic individuals. Within this cohort, we included patients who underwent testing for both CRP and PCT on at least one occasion during their hospital admission. Testing of CRP was done according to the discretion of the clinician and commonly performed once daily across care settings. A baseline PCT was sent depending on clinical suspicion of bacterial co-infection, and repeated at 24–48 hourly intervals as appropriate. Concentrations of CRP and PCT were analysed using a chemiluminescence and turbidimetry method respectively (Abbott, USA) in UKAS accredited laboratories.

We identified 237 patients who were included in our study. Microbiology results from blood, urine and respiratory tract, which grew organisms deemed pathogenic and significant by the clinical and microbiology team were included, excluding potential contaminants such as *Coagulase negative Staphylococcus*. All patient data from an initial 3-week period from the admission date were anonymised and extracted to a database for analysis.

Statistical testing was conducted in R (R Foundation, Austria). Longitudinal analysis was set out in two steps over a pre-processed dataset from patient electronic health records. Firstly, to examine longitudinal data at different time horizons we segmented aggregate patient data into time windows conferring to periods of clinical interest [10]. These time series windows were subsequently used to determine the difference in biomarker levels across time between patient groups through a Mann–Whitney test. Secondly, we analysed patient results in the form of trajectories of biomarkers over time. Rate of change over varying time horizons was computed by fitting a series of linear models to individual trajectories [11]. Statistical significance was adjusted for multiple testing through Bonferroni correction in the longitudinal analyses to give a significance threshold of p < 0.001 or lower depending on the number of tests performed in the series.

## Results

Baseline characteristics of the 237 patients included in analyses, stratified by in-hospital mortality are displayed in Table 1. The median age was 67 years old (Inter quartile range, IQR 54–79) and around two-thirds of admitted patients during this period were male (144/237, 60.8%). The median day of illness on hospital presentation was 7 days (IQR 3–10) and median length of stay for patients was 9 days (IQR 5–18). Fifty-nine patients (24.9%) were admitted to the intensive care unit (ICU) for level 2 or 3 organ support, namely single or multiple organ support such as renal, cardiovascular or invasive ventilatory support; patients who were cared for on general wards received supplementary oxygenation up to 15 L/min but no additional organ support.

**Table 1:** Clinical characteristics and microbiology culture results of included patients stratified by in-hospital mortality

Category	All patients (n = 237)	Survived (n = 182)	Died (n = 55)	p-value
Median age in years (IQR)	67 (54–79)	66 (51–78)	69 (60–81)	NS
Male	144 (60.8%)	105/144 (72.9%)	39/144 (27.1%)	NS
Female	93 (39.2%)	77/93 (82.8%)	16/93 (17.2%)	
Received level 2 or level 3 care	59 (24.8%)	34/182 (18.7%)	25/55 (45.5%)	< 0.0001
Median presenting day of illness (IQR)	7 (3–10)	7 (3–11)	7 (3–10)	NS
Median length of stay in hospital in days (IQR)	9 (5–18)	8 (4–17)	10 (7–20)	NS
Median CRP concentration on days 1–2 of admission		125 mg/L	241 mg/L	< 0.0001
Median PCT concentration on days 1–2 of admission		0.13 ng/mL	0.245 ng/mL	NS
Bacterial cultures taken once or more times during admission from:				
Blood—203/237 (86%) patients sampled	14/203 (6.9%) positive	11 (6.0%)	3 (5.5%)	NS
Urine—123/237 (52%) patients sampled	21/123 (17.1%)	18 (9.9%)	3 (5.5%)	NS
Respiratory tract—62/237 (27%) patients sampled	24/62 (38.7%)	17 (9.3%)	7 (12.7%)	NS

A p-value threshold of > 0.05 was denoted as statistically insignificant (NS)

The majority of patients in the cohort (87%, 207/237) underwent microbiological sampling (blood, urine or respiratory) at least once during admission, of which 28% (58/207) had at least one clinically significant microbiology result during admission. The median day of microbiological sampling for positive cultures was 7 days (IQR 1–14) from admission. The most common isolate from blood (excluding potential skin contaminants) was *Staphylococcus aureus* (4/14 patients with positive samples), *Escherichia coli* was most commonly isolated from urine (13/21 patients) and *Klebsiella pneumoniae* or *Pseudomonas aeruginosa* (7/24 patients for both) from the respiratory tract. Patients admitted to the intensive care department were more likely to have positive cultures than patients remaining in general care (60.3 vs 24.8% respectively, p < 0.001). However, there was no significant difference in rates of positive cultures between patients who survived or died (32.1 vs 34.9% respectively, p = 0.7, see Additional file 1: Table S1).

**Fig. 1:**
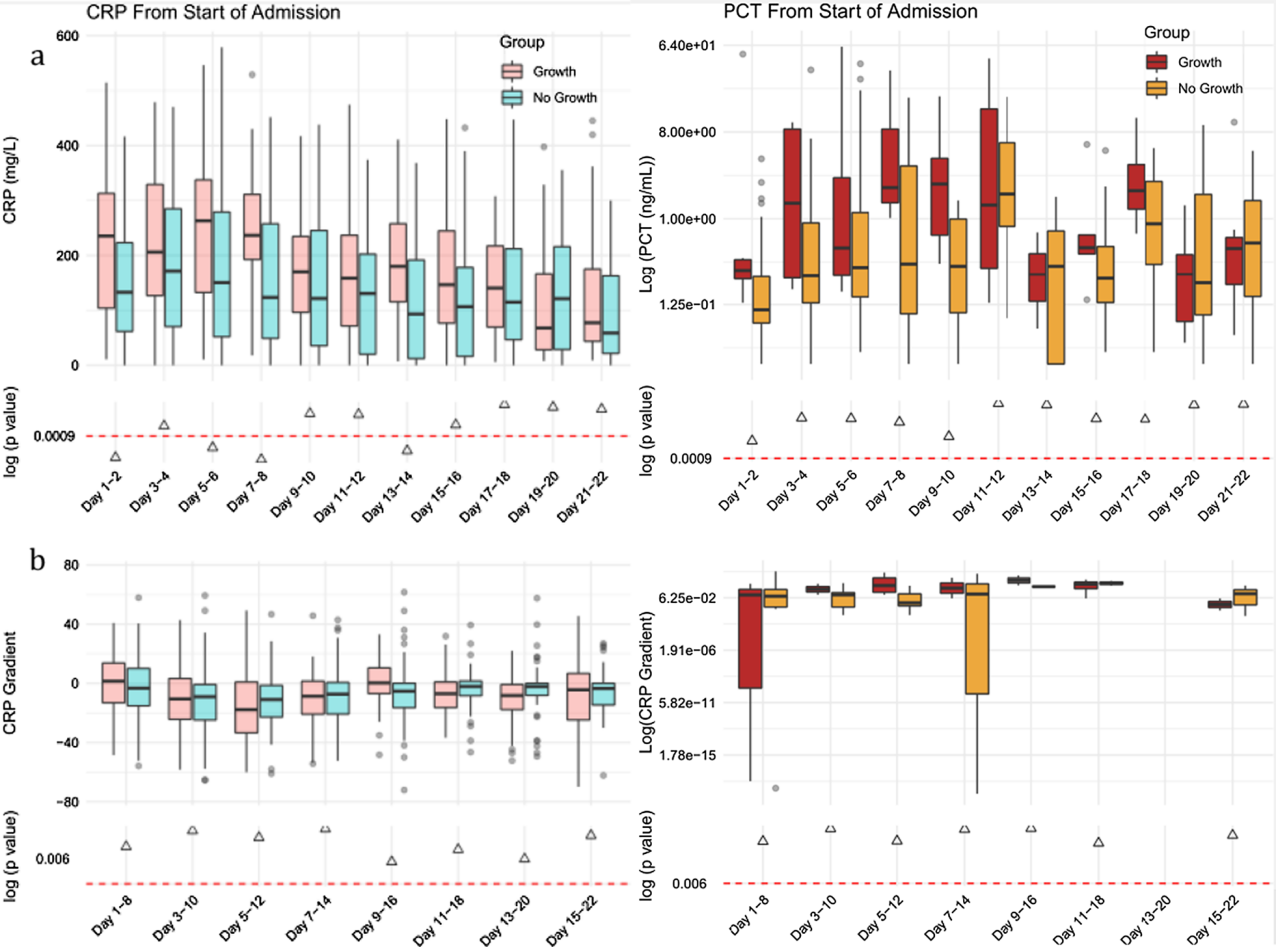
**a** Longitudinal trend of CRP and PCT concentrations and **b** the rate of change from aggregated median values grouped by microbiological culture positivity for any of: blood/urine/respiratory tract. The dotted p-value line denotes statistical significance threshold corrected for multiple testing and triangles below the line represent p < 0.001 for a particular pairwise comparison

**Fig. 2:**
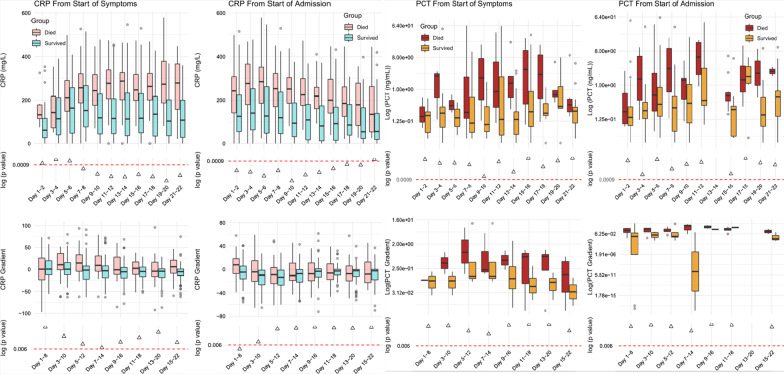
Longitudinal trend of CRP and PCT concentrations and rate of change from aggregated median values grouped by in hospital death or survival. Two time series alignments were performed **a** alignment by days from start of symptoms, and **b** alignment from start of admission

Aggregated median values of 2,021 data points for CRP and 284 points for PCT were plotted, stratified against the day of illness and admission with respect to positive microbiological cultures (Fig. 1) or survival up to week 3 of admission (Fig. 2). We show a difference in aggregated CRP concentrations over time in those with positive versus negative microbiology, most significant before day 9 (236 vs 123 mg/L, p < 0.0001). There was also a significant difference in CRP between those who died or survived, significant only after 7–8 days of symptom onset (226 vs 152 mg/L, p < 0.0001) and this difference was sustained up to 21 days after admission. Moreover, the rate of change in CRP between days 1 and 8 post admission exhibited a significantly negative trend in patients who survived (− 4.86 vs + 7.7 during day 1–8 window, p = 0.002). When patients were stratified according to the place of care (general ward vs non-ICU settings)—we show that CRP concentrations were consistently higher throughout admission in those who had positive microbiology as well as in those who died (Additional file 2: Fig. S1).

For PCT, concentrations over time were consistently higher in patients with positive microbiology against those without (concentrations on days 7–8, 2.11 vs 0.28 ng/mL), although this did not reach statistical significance (p = 0.08, Fig. 1). There was also a trend towards higher PCT levels in patients who died, but again differences over time were not statistically significant (Fig. 2).

## Discussion

In a cohort of hospitalised patients with COVID-19, we show that CRP concentrations were significantly higher in patients with positive microbiological cultures as well as in patients who died. There was also a specific 7-day window from admission (corresponding to days 8–14 of symptom onset), during which lower concentrations of CRP were associated with survival. Changes in CRP concentration seen in COVID-19 are likely reflective of robust inflammatory responses and cytokine release [12], however, our results indicate their utility to also predict the presence of significant microbiology and potential bacterial coinfection. We show that understanding disease timing and changes in CRP concentration are important in utilising biomarker information effectively. This is particularly relevant for patients presenting with early illnesses such as those encountered in community and pre-hospital settings.

Procalcitonin has been investigated as a biomarker to guide antimicrobial use, through differentiating between viral and bacterial infections particularly in sepsis and pneumonia. The utility of PCT to guide antibiotic therapy in COVID-19 however remains unclear, given conflicting data and continued clinical research remains a priority [13]. There is support that PCT can be non-specifically elevated as a function of clinical severity in pneumonia regardless of aetiology [14]. We observed a difference in median PCT for microbiological culture positivity and survival. These differences, which were not statistically significant, may however be due to the relatively fewer data points available compared to CRP, leading to unbalanced data and use of Bonferroni correction to account for multiple testing, which can be overly conservative. Nonetheless, the relatively low PCT (< 0.5 ng/mL) concentration and absence of significance between outcome groups in the first 48 h of admission might suggest a low burden of community-acquired bacterial coinfection in patients presenting with COVID-19, consistent with other studies [15].

Our analysis is novel in the inclusion of a large biomarker dataset and modelling of their change over time in addition to comparing their static concentrations at defined clinical time points. Accounting for these biomarker dynamics when coupled with relevant clinical assessments could support the de-escalation or cessation of antimicrobials in COVID—particularly at a time when both the probability of bacterial coinfection and severity are deemed to be low. Development of such systems will be crucial given empirical antimicrobial use in the management of COVID is high and assessment of co-infection particularly challenging [16]. Likewise, novel methods of patient monitoring, such as biosensors capable of continuous monitoring of markers such as CRP, procalcitonin or lactate may have a role [17]. Taking into account the changing patient risks over time reflects real-world clinical decision making, and examination of interactions between an extended number of biomarkers with clinical data may be particularly suited through artificial intelligence approaches. We have previously shown that such an approach which uses machine learning to guide antimicrobial use for suspected bacterial infections performed effectively in clinical evaluation [18]. Other strengths of the study include use of a real-world cohort, employment of robust modelling techniques, the large number of data points included and the longitudinal approach. We limited our analyses to a short time window, focussing on objective patient outcomes and performed day-by-day comparisons between groups to limit survivor bias.

Limitations include the retrospective nature of our analysis of a heterogenous groups of patients cared for in intensive care and ward environments, which may have introduced a selection bias: for example, patients admitted to intensive care might have been sampled more frequently. Patients who were critically ill may also be deemed clinically unsuitable for escalation of care and contribute to result bias. We used positive microbiological culture results as a pragmatic indicator of bacterial infection: differentiating true infection from colonisation can be difficult as the majority of hospitalised patients present with fever, high inflammatory markers and require oxygenation. However in most cases the additional information on positive bacterial isolates were treated as clinically significant by the medical team and managed accordingly. The limitations in sensitivity for microbiological culture methods to detect presence of infection is also acknowledged.

There was a higher overall incidence of positive bacterial isolation in this study in comparison with other pooled estimates (28 vs 14.3%) [19]. Inclusion into this cohort was selected on the basis of having data on CRP and PCT, and this could bias the cohort in favour of patients at higher risk of bacterial infection. There was also a high rate of microbiological sampling (87% received one or more bacterial cultures during admission), but these factors nonetheless emphasise the need to formalise definitions of bacterial coinfection and secondary infections for COVID-19 [20] in order to understand the true incidences. Biomarker sampling in patients, particularly of PCT, was guided mainly according to clinical suspicion of bacterial co-infection. At the time of the study, consensus on the use of PCT specifically in the context of COVID-19 have not been reached leading to individual variation.

## Conclusion

In conclusion, we show that the dynamic changes in CRP concentrations over time can be predictive of both, microbiological and survival outcomes in the context of COVID-19 in our patient group. Use of these findings in a risk model coupled with stewardship input and use of rapid diagnostics could guide and support a formalised avenue in optimising antimicrobial management for COVID-19.

## Supplementary Information

**Additional file 1: Table S1.** Details of positive microbiology cultures by site of sampling and patient outcome. Numbers (n) denote patients who had a positive microbiology result for that particular organism during hospital admission

**Additional file 2: Fig. S1.** Longitudinal CRP concentrations plotted against day of admission (left) or day of symptom onset (right) for patients managed in intensive care unit or level 1 settings (n=69) or general ward settings (n=168). Figure 1 denotes CRP concentration stratified by in-hospital mortality with red showing patients who died and blue showing patients alive. Figure 2 denotes CRP concentration stratified by positive or negative microbiology during hospital admission 

## Data Availability

This is available on request – please contact the corresponding author.

## Abbreviations

CRP: C-reactive protein
PCT: Procalcitonin
COVID-19: Coronavirus disease 2019
IQR: Interquartile range
ICU: Intensive care unit
SARS-COV-2: Severe acute respiratory syndrome coronavirus 2
WHO: World Health Organisation

## References

[1] Lansbury L, Lim B, Baskaran V, Lim WS (2020). Co-infections in people with COVID-19: a systematic review and meta-analysis. J Infect.

[2] Zhou F, Yu T, Du R, Fan G, Liu Y, Liu Z (2020). Clinical course and risk factors for mortality of adult inpatients with COVID-19 in Wuhan, China: a retrospective cohort study. Lancet.

[3] Rawson TM, Ming D, Ahmad R, Moore LSP, Holmes AH (2020). Antimicrobial use, drug-resistant infections and COVID-19. Nat Rev Microbiol.

[4] Rawson TM, Moore LSP, Castro-Sanchez E, Charani E, Davies F, Satta G (2020). COVID-19 and the potential long-term impact on antimicrobial resistance. J Antimicrob Chemother.

[5] World Health Organisation. Clinical management of COVID-19 - interim guidance. 2020. https://www.who.int/publications/i/item/clinical-management-of-covid-19

[6] Simon L, Gauvin F, Amre DK, Saint-Louis P, Lacroix J (2004). Serum procalcitonin and C-reactive protein levels as markers of bacterial infection: a systematic review and meta-analysis. Clin Infect Dis.

[7] Wang D, Hu B, Hu C, Zhu F, Liu X, Zhang J (2020). Clinical characteristics of 138 hospitalized patients with 2019 novel coronavirus-infected pneumonia in Wuhan, China. JAMA.

[8] Liu F, Li L, Xu M, Wu J, Luo D, Zhu Y (2020). Prognostic value of interleukin-6, C-reactive protein, and procalcitonin in patients with COVID-19. J Clin Virol.

[9] Luo X (2020). Prognostic value of C-reactive protein in patients with COVID-19. Clin Infect Dis.

[10] Sherman E, Gurm H, Balis U, Owens S, Wiens J (2017). Leveraging clinical time-series data for prediction: a cautionary tale. AMIA Annu Symp Proc.

[11] Hastie T, Tibshirani R, Friedman JH (2001). The elements of statistical learning: data mining, inference, and prediction.

[12] Qin C, Zhou L, Hu Z, Zhang S, Yang S, Tao Y (2019). Dysregulation of immune response in patients with coronavirus 2019 (COVID-19) in Wuhan, China. Clin Infect Dis.

[13] National Institute for Health and Care Excellence. COVID-19 rapid guideline: antibiotics for pneumonia in adults in hospital. 2020. 33400459

[14] Gautam S, Cohen AJ, Stahl Y, Toro PV, Young GM, Datta R (2020). Severe respiratory viral infection induces procalcitonin in the absence of bacterial pneumonia. Thorax.

[15] Garcia-Vidal C, Sanjuan G, Moreno-García E, Puerta-Alcalde P, Garcia-Pouton N, Chumbita M (2020). Incidence of co-infections and superinfections in hospitalised patients with COVID-19: a retrospective cohort study. Clin Microbiol Infect.

[16] Huttner BD, Catho G, Pano-Pardo JR, Pulcini C, Schouten J (2020). COVID-19: don’t neglect antimicrobial stewardship principles!. Clin Microbiol Infect.

[17] Bollella P, Sharma S, Cass AEG, Antiochia R (2019). Microneedle-based biosensor for minimally-invasive lactate detection. Biosens Bioelectron.

[18] Rawson TM, Hernandez B, Moore LSP, Herrero P, Charani E, Ming D (2020). A real-world evaluation of a case-based reasoning algorithm to support antimicrobial prescribing decisions in acute care. Clin Infect Dis.

[19] Langford BJ, So M, Raybardhan S, Leung V, Westwood D, MacFadden DR (2020). Bacterial co-infection and secondary infection in patients with COVID-19: a living rapid review and meta-analysis. Clin Microbiol Infect.

[20] Rawson TM, Moore LSP, Zhu N, Ranganathan N, Skolimowska K, Gilchrist M (2020). Bacterial and fungal co-infection in individuals with coronavirus: a rapid review to support COVID-19 antimicrobial prescribing. Clin Infect Dis.

