# Correction to: Informing antimicrobial management in the context of COVID-19: understanding the longitudinal dynamics of C-reactive protein and procalcitonin

**DOI:** 10.1186/s12879-021-06696-2

**Published:** 2021-09-21

**Authors:** Damien K. Ming, Ashleigh C. Myall, Bernard Hernandez, Andrea Y. Weiße, Robert L. Peach, Mauricio Barahona, Timothy M. Rawson, Alison H. Holmes

**Affiliations:** 1 Centre for Antimicrobial Optimisation, Hammersmith Hospital, Imperial College London, Du Cane Road, London, W12 0NN UK; 2 National Institute for Health Research Health Protection Research Unit in Healthcare Associated Infections and Antimicrobial Resistance, Imperial College London, Hammersmith Campus, Du Cane Road, London, W12 0NN UK; 3 School of Informatics, University of Edinburgh, Scotland, UK; 4 School of Biological Science, University of Edinburgh, Scotland, UK; 5 Department of Neurology, University Hospital of Würzburg, 97080 Würzburg, Germany; 6 Department of Mathematics, Imperial College London, London, UK

## Correction to: BMC Infect Dis (2021) 21:932 10.1186/s12879-021-06621-7

Following publication of the original article [1], the authors identified an error in the author name of Bernard Hernandez.

The incorrect author’s name is: Bernarnd Hernandez;

The correct author’s name is: Bernard Hernandez;

The author group has been updated above and the original article [1] has been corrected.
