# Supplementary material for: Informing antimicrobial management in the context of COVID-19: understanding the longitudinal dynamics of C-reactive protein and procalcitonin
Source: BMC Infect Dis. 2021 Sep 8;21:932. doi: 10.1186/s12879-021-06621-7 (PMC8424157; doi:10.1186/s12879-021-06621-7)
Supplement: Supplementary file 1 — Additional file 1: Table S1. Details of positive microbiology cultures by site of sampling and patient outcome. Numbers (n) denote patients who had a positive microbiology result for that particular organism during hospital admission [file 12879_2021_6621_MOESM1_ESM.docx]

| **Type of culture** | **Organism** | **All (n)** | **Alive (n)** | **Died (n)** |
| --- | --- | --- | --- | --- |
| Blood culture: 203 patients sampled | *Staphylococcus aureus* | 4 (29%) | 4 | 0 |
|  | *Streptococcus spp.* | 2 (14%) | 2 | 0 |
|  | *Klebsiella pneumoniae* | 3 (21%) | 1 | 2 |
|  | Others | 5 (36%) | 4 | 1 |
|  | Total | 14 | 11 | 3 |
| Urine culture: 123 patients sampled | *Escherichia coli* | 13 (62%) | 11 | 2 |
|  | *Klebsiella pneumoniae* | 3 (14%) | 3 | 0 |
|  | *Enterobacter cloacae* | 2 (10%) | 2 | 0 |
|  | *Pseudomonas aeruginosa* | 2 (10%) | 2 | 0 |
|  | Others | 1 (5%) | 0 | 1 |
|  | Total | 21 | 18 | 3 |
| Respiratory culture: 62 patients sampled | *Klebsiella pneumoniae* | 7 (29%) | 4 | 3 |
|  | *Pseudomonas aeruginosa* | 7 (29%) | 6 | 1 |
|  | *Stenotrophomonas spp.* | 3 (13%) | 3 | 0 |
|  | *Escherichia coli* | 3 (13%) | 2 | 1 |
|  | Others | 4 (17%) | 2 | 2 |
|  | Total | 24 | 17 | 7 |

**Supplementary table 1.** Details of positive microbiology cultures by site of sampling and patient outcome. Numbers (n) denote patients who had a positive microbiology result for that particular organism during hospital admission.
