# Supplementary material for: Informing antimicrobial management in the context of COVID-19: understanding the longitudinal dynamics of C-reactive protein and procalcitonin
Source: BMC Infect Dis. 2021 Sep 8;21:932. doi: 10.1186/s12879-021-06621-7 (PMC8424157; doi:10.1186/s12879-021-06621-7)
Supplement: Supplementary file 2 — Additional file 2: Fig. S1. Longitudinal CRP concentrations plotted against day of admission (left) or day of symptom onset (right) for patients managed in intensive care unit or level 1 settings (n=69) or general ward settings (n=168). Figure 1 denotes CRP concentration stratified by in-hospital mortality with red showing patients who died and blue showing patients alive. Figure 2 denotes CRP concentration stratified by positive or negative microbiology during hospital admission [file 12879_2021_6621_MOESM2_ESM.docx]

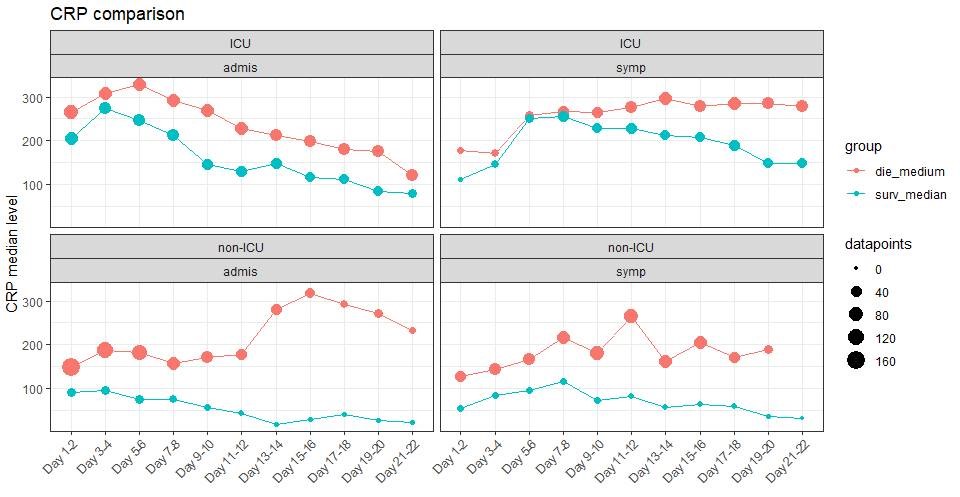

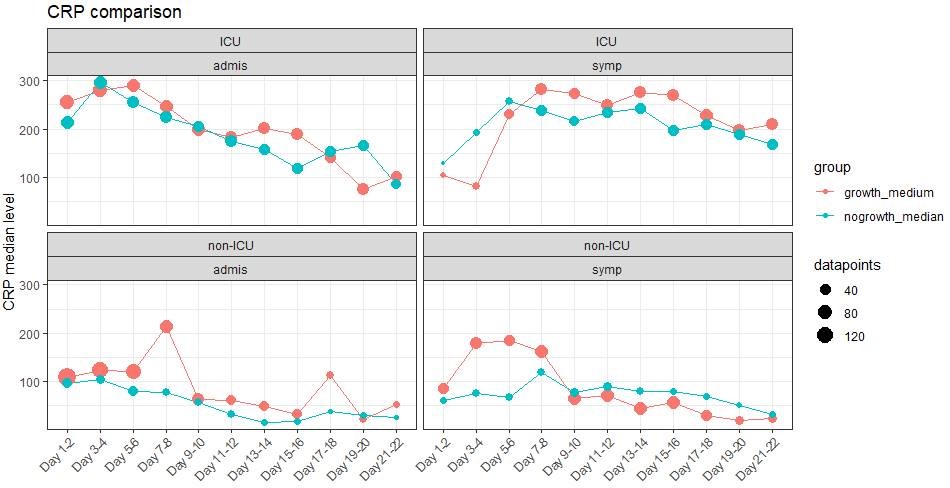


2

1

**Supplementary figure.** Longitudinal CRP concentrations plotted against day of admission (left) or day of symptom onset (right) for patients managed in intensive care unit or level 1 settings (n=69) or general ward settings (n=168). Figure 1 denotes CRP concentration stratified by in-hospital mortality with red showing patients who died and blue showing patients alive. Figure 2 denotes CRP concentration stratified by positive or negative microbiology during hospital admission.
